# Olive cultivar origin is a major cause of polymorphism for Ole e 1 pollen allergen

**DOI:** 10.1186/1471-2229-8-10

**Published:** 2008-01-25

**Authors:** AbdelMounim Hamman-Khalifa, Antonio Jesús Castro, José Carlos Jiménez-López, María Isabel Rodríguez-García, Juan de Dios Alché

**Affiliations:** 1 Department of Biochemistry, Cell and Molecular Biology of Plants, Estación, Experimental del Zaidín, Consejo Superior de Investigaciones Científicas (CSIC), Profesor Albareda 1, 18008, Granada, Spain

## Abstract

**Background:**

Pollens from different olive (*Olea europaea *L.) cultivars have been shown to differ significantly in their content in Ole e 1 and in their overall allergenicity. This allergen is, in addition, characterized by a high degree of polymorphism in its sequence. The purpose of this study is to evaluate the putative presence of divergences in Ole e 1 sequences from different olive cultivars.

**Results:**

RNA from pollen individually collected from 10 olive cultivars was used to amplify Ole e 1 sequences by RT-PCR, and the sequences were analyzed by using different bioinformatics tools. Numerous nucleotide substitutions were detected throughout the sequences, many of which resulted in amino acid substitutions in the deduced protein sequences. In most cases variability within a single variety was much lower than among varieties. Key amino acid changes in comparison with "canonical" sequences previously described in the literature included: a) the substitution of C19-relevant to the disulphide bond structure of the protein-, b) the presence of an additional N-glycosylation motif, and c) point substitutions affecting regions of Ole e 1 already described like relevant for the immunogenicity/allergenicity of the protein.

**Conclusion:**

Varietal origin of olive pollen is a major factor determining the diversity of Ole e 1 variants. We consider this information of capital importance for the optimal design of efficient and safe allergen formulations, and useful for the genetic engineering of modified forms of the allergen among other applications.

## Background

Olive (*Olea europaea *L.) pollen produces respiratory allergy in the populations of geographical areas where this plant is widely cultivated, mainly the countries surrounding the Mediterranean Sea and several regions of North, South America and Australia [1,2]. Up to date, 10 allergens, i.e. Ole e 1 to Ole e 10, have been purified and characterized in *Olea europaea *L. pollen [3,4]. The major allergen Ole e 1 is a protein of 18–22 kDa, displaying acidic pI and different forms of N-glycosylation [5,6]. Besides its high homology to its counterparts in other members of the *Oleaceae *family, such as lilac and privet [7,8], Ole e 1 exhibits relevant similarity with the products of genes Lat52 from tomato, Zm13 from maize and several extensins from *Arabidopsis thaliana *and *Nicotiana tabacum *among other proteins. These proteins have been indexed like members of the denominated "pollen proteins of the Ole e 1 family" (Accession number: PF01190) within the Pfam protein families database [9]. These plant pollen proteins are structurally related. They are most probably secreted and consist of about 145 residues. There are 6 cysteines conserved in the sequence of these proteins which seem to be involved in disulphide bonds. Although their biological function is not yet known, they have been suggested to be involved in important events during pollen formation, such as hydration, germination and/or pollen tube growth [10-13].

Pollen from different olive cultivars considerably differ in their capacity to bind IgE antibodies and to elicit an allergenic response, as tested by different *in vivo *and *in vitro *techniques [14-17]. Noteworthy, quantitative differences in the content of Ole e 1 have been described in the pollen of several olive cultivars, which are positively correlated with the amounts of Ole e 1 transcripts present in these pollens [17]. Although these quantitative differences are likely responsible for the different reactivity of patients to the pollen from different cultivars, the concomitances of other factors can not be discarded. In this context, the role of other major and secondary allergens from olive pollen, and the presence of divergent epitopes of Ole e 1 in the different cultivars should be evaluated.

Ole e 1 presents numerous microheterogeneities in its nucleotide sequence [18,19]. These nucleotide substitutions result in many cases in amino acid changes in the natural protein [20], which have been reported to influence the binding of IgE molecules to Ole e 1 proteins [21]. However, up to date, the presence of such polymorphism has never been related to the genetic origin of the pollen tested. On the contrary, commercially available pollen from uncertain origin or pollen mixes from different sources seems to be the common material used for biochemical, molecular and immunological characterization of allergens. The presence of an extremely wide germplasm in the olive, with over 1200 varieties cultivated over the world [22] clearly point to this genetic variability as a putative cause of inconsistency for Ole e 1 sequence. This work explores the molecular variability of Ole e 1 allergen throughout a number of olive cultivars and discusses putative implications of such polymorphism in both the biology of the plant and the development of the allergic symptoms.

## Results

### Sequences analysis

Ole e 1 RT-PCR amplification of RNA from pollen of eight olive cultivars, and further sequencing of three clones from each cloned PCR product resulted in twenty four raw sequences. The nucleotide sequences obtained were deposited in the GenBank™/EMBL Database (Table 1).

**Table 1:** GenBank™/EMBL Database entries of the Ole e 1 cDNA sequences obtained.

**Cultivar/Clone**	**Accession No.**
Picholine 1	AF500908
Picholine 2	AF515277
Picholine 3	AF515278
Menara 1	AF515279
Menara 2	AF515280
Menara 3	AF515281
Lucio 1	AY137467
Lucio 2	AY137468
Lucio 3	AY137469
Picual 1	AF532760
Picual 2	AF532753
Picual 3	AF532754
Loaime 1	AF532755
Loaime 2	AF532756
Loaime 3	AF532757
Hojiblanca 1	AF532758
Hojiblanca 2	AF532761
Hojiblanca 3	AF532762
Arbequina 1	AF532759
Arbequina 2	AF532763
Arbequina 3	AF532764
B. España 1	AF532765
B. España 2	AF532766
B. España 3	AF532767

These sequences were individually analyzed by the nucleotide-nucleotide BLAST (blastn) program [23]. Higher identity scores included in all cases the different variants of Ole e 1 present in the nr (non-redundant All GenBank+EMBL+DDBJ+PDB sequences – but no EST, STS, GSS, environmental samples or phase 0, 1 or 2 HTGS sequences-) database, including the following accessions: X76396.1, X76397.1, Y12426.1, X76395.1, AY159880.1, S75766.1, Y12428.1 and Y12427.1. Significant scores (E. value threshold: 2e-99) also corresponded to Ole e 1-like proteins from *Ligustrum vulgare *(X77788.1, X77787.1), *Fraxinus excelsior *(AF526295.1, AY652744.1, AY377127.1), *Syringa vulgaris *(X76539.1, X76540.1, X76541.1) and wild olive -acebuche- (AY159881.1). The identity analysis involved the whole sequence of these variants, with the exception of a short 5' fragment of 38–44 nucleotides including the ATG initiation codon which was absent in the RT-PCR amplified fragments as the result of the amplification strategy used.

**Figure 1:**
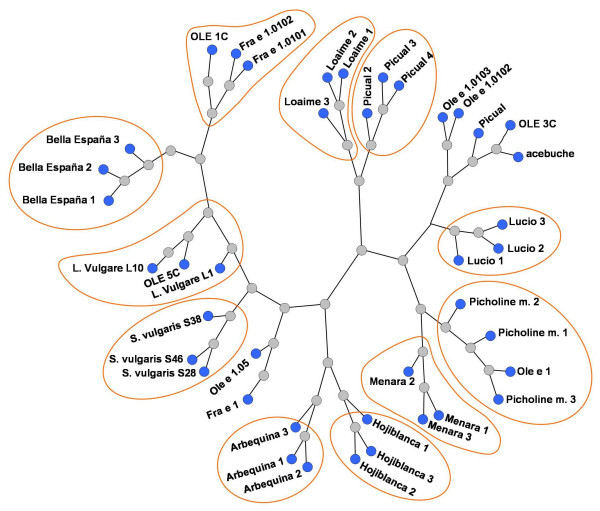
Tree view corresponding to the higher scores (E. value threshold: 2e-99) of a BLAST pairwise alignment of the submitted Ole e 1 sequences within the nr databases. Ole e 1 sequences of the same cultivar origin are frequently grouped in the same branch of the tree. Distances among some olive cultivars (i.e. Picual and Bella de España) were in some cases larger than between olive cultivars and Ole e 1-like proteins from members of other genera of the *Oleaceae *family (i.e. Bella de España and *Fraxinus*/*Ligustrum*).

Different tree views were computed based on BLAST pairwise alignment of the query sequences to the sequences searched in the databases (see Fig. 1 as an example). The tree views generated clearly displayed that sequence differences were appreciably higher among olive cultivars than within the same cultivar. Differences between some cultivars (i.e. Picual and Bella de España) were in some cases higher than between olive cultivars and Ole e 1-like proteins from members of the *Oleaceae *family.

**Figure 2:**
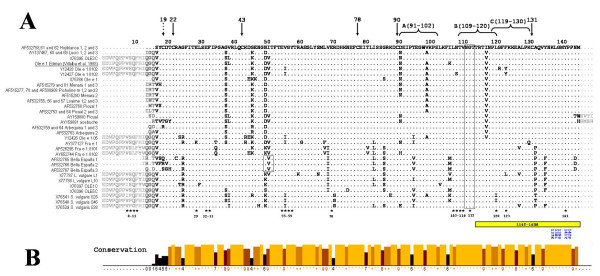
**A: **Multiple alignment of the deduced Ole e 1 amino acid sequences displayed in Figs 1, Additional file 1, and the amino acid sequence of Ole e 1 experimentally determined by Edman degradation (20). Conserved Cys at positions 22, 43, 78, 90 and 131 are pointed by arrows (discontinuous line arrow for the semi-conserved 19C). Predicted N-glycosylation motifs are indicated by boxes. Immunodominant T-cell epitopes (A, B and C), experimentally determined by [24] are showed by brackets. IgE and IgG B-cell immunodominant regions of Ole e 1, determined by [25] are respectively shown by stars and bars. Numbering follows the experimental Ole e 1 sequence obtained by Edman degradation. **B: **Sequence conservation index calculated by Jalview programme according to [53]. Conservation is measured as a numerical index (0 to 10) reflecting the conservation of physico-chemical properties for each column of the alignment: identities score highest, and the next most conserved group contain substitutions to amino acid lying in the same physico-chemical class.

Detailed analysis of sequences was performed after multiple alignment of the 24 nucleotide sequences obtained, using ClustalW software. The analysis revealed the presence of microheterogeneities at several positions, particularly in the 5' coding region and the 3' non-coding region [see Additional file 1]. However, the main variability was found in the sequences corresponding to Bella de España, in which a fragment of 38 nucleotide was absent from the 3' non-coding region. The reported changes in many cases also affected the corresponding deduced amino acid sequences, as displayed in Figure 2.

The numerical analysis of the nucleotide and amino acid substitutions (including conservative substitutions) detected among the sequences obtained and those showing a relatively high percentage of identity is displayed in Additional file 2. The percentage of identical nucleotides between the Ole e 1 sequences ranged between 86 to 100%, whereas the percentage of identical nucleotides between Ole e 1 and Ole e 1-like sequences from other *Oleaceae *members ranged between 86 to 93%.

When the predicted amino acid sequences were analyzed, the percentage of identical amino acids ranged between 82 and 100% among the Ole e 1 sequences (84 to 96% between Ole e 1 and Ole e 1-like sequences from other *Oleaceae *members). After considering conservative changes, these percentages raised into ranges of 90–100% for Ole e 1 sequences, and 90–98% between Ole e 1 and Ole e 1-like sequences.

Amino acid substitutions were spread over the whole Ole e 1 sequence with the exception of several regions, which were extremely conserved throughout all the sequences analyzed, independently of the variety of origin (Figure 2). These conserved regions included those corresponding to 5 out of the 6 Cys conserved residues at positions 22, 43, 78, 90 and 131, and a putative N-glycosylation site at position 111.

Several highly conserved sequences also corresponded to immunodominant T-cell epitopes of Ole e 1 allergen characterized by [24] and amino acid residues implicated in IgG and IgE binding [25]. Some of the predicted sequences were identical to that described by [20], which was obtained for Ole e 1 after Edman degradation. These identical sequences were obtained from cultivars Lucio, Menara and Picholine marocaine, in addition to the sequence S75766, which was previously obtained in our laboratory from an undetermined source of pollen.

On the other hand, the analysis using different bioinformatic tools and those sequences described as relevant for the allergenic response [24,25] allowed us to identify key amino acid substitutions in the sequences corresponding to several cultivars, which are listed in Additional file 3. These substitutions included: a) Cys residues substituted at position 19 in 6 sequences (3 from the cultivar Bella de España, 2 from the cultivar Arbequina, and that corresponding to the wild olive -acebuche-) b) an extra site for potential N-glycosylation at position 50, predicted by the ScanProsite software within the three amino acid sequences obtained from the cultivar Bella de España and c), point substitutions in those sequences described like immunodominant T-cell epitopes of Ole e 1 allergen characterized by [24] and amino acid residues implicated in IgG and IgE binding [25].

## Discussion

Polymorphism is a frequent feature for allergens molecules. Different degrees of polymorphism have been described in the allergens of many different sources, including house dust mites [26], foods [27,28] and allergens from different grass and tree pollens [29,30]. However, although allergen polymorphism is beginning to be deeply characterized at the molecular level, little is known in many cases regarding the origin of such polymorphism. In several cases, allergen polymorphism has been attributed to the presence of multigene protein families [31]. In other allergens, the presence of post-translational modifications may also determine the presence of multiple forms of the allergen, as is the case of Ole e 1 [32]. In apple (*Malus domestica*), up to 18 Mal d 1 genes have been characterized. Allelic diversity regarding this allergen has been considered as a major explanation for the considerable differences in allergenicity, widely described among apple cultivars [28]. The high rate of nucleotide substitutions detected in Ole e 1 indicates that these substitutions are unlikely to represent *in vitro *artefacts as the result of error incorporations by the Pfu polymerase used. The present paper demonstrates that Ole e 1 polymorphism is clearly linked to the cultivar (genetic) origin of the pollen analyzed, and therefore represents the basis for further research in relation to the biological function and the allergenicity of this gene product.

It was previously reported by us [17] that pollen from different olive cultivars exhibit quantitative differences in the content of Ole e 1, which were positively correlated with the amounts of Ole e 1 transcripts present in these pollens. The results shown here also indicate that qualitative differences in the Ole e 1 allergen also occur in these pollens. Three major parameters have been demonstrated to influence the reactivity of Ole e 1 to IgG and IgE antibodies in a higher or lower degree [21]: three-dimensional (3D)-folding, the glycan component and several point changes of the amino acid sequence. The present paper reports, on the basis of the sequences obtained, that modifications in these features can be related to the genetic origin of the pollen analyzed.

Sequence substitutions in Ole e 1 from different varieties are not equally distributed over the whole sequence. Conserved regions include those corresponding to the conserved Cysteine residues at positions 22, 43, 78, 90 and 131. The absence up to date of a 3D model for Ole e 1 or any Ole e 1-like protein with significant identity to this protein within the PDB databases makes impossible to determine precisely the putative involvement of amino acid substitutions into conformational changes of the protein, which may affect either the biological function or the IgG/IgE binding ability of the protein. However, we may speculate about the potential changes. Substitution of 19C may dramatically alter tertiary structure of the Ole e 1 protein, as the presence of a disulphide bond between 19C–90C in Ole e 1 was previously established [33]. The conformation proposed by these authors is supposed to be shared by most members of proteins of the Ole e 1 family, where an identical distribution of the 6 cysteine residues is widely maintained, no matter their phylogenetic distances or relationships. As a consequence, important modifications in the reactivity of antibodies to conformational epitopes might be expected, as the binding of Ole e 1 to human IgE antibodies has been shown to be highly dependent on the integrity of the disulphide bridges [21]. The putative absence of the 19C–90C disulfide bridge in some Ole e 1 forms, could also represent an additional hint against the proposed homology between the Kunitz protein family and Ole e 1-related pollen proteins [34,35], since disulfide arrangement is a typical feature for the classification of the protease inhibitor protein family [36]. The presence of free – SH groups (corresponding to the 90C) in these Ole e 1 forms with substitutions in the 19C residue, and therefore unable to form the 19C–90C intramolecular bridge, could alternatively promote the formation of dimeric forms of the protein throughout the formation of intermolecular disulphide bridges. It has been described that the SDS-PAGE pattern of Ole e 1 frequently exhibits a faint band of 40 kDa as a result of the presence of a 20 kD dimer [20], which may account up to 5% of Ole e 1. Our observations show that these dimeric forms of Ole e 1 are relatively more abundant in the extracts of Bella de España and Arbequina than in the rest of cultivars examined. Monomeric and dimeric forms of Ole e 1 also react differentially to a panel of monoclonal antibodies to Ole e 1 and to sera from olive-allergic patients (unpublished results).

The role of the glycan moiety of Ole e 1 in antibody binding has also been established [6,7,21,37]. These authors concluded that carbohydrate moieties may constitute determinants possessing a relative significance in the binding of IgE from hypersensitive patients, not only in the Ole e 1 allergen but also in other allergenic glycoproteins. In this context, the presence of modifications, such as the putative presence of an extra site for N-glycosylation which is reported here for the cultivar Bella de España may represent an important modification in the allergenic capability of the Ole e 1 protein of this pollen, which should be further tested.

Several examples are available throughout the literature demonstrating that sequence changes need not be extensive to significantly alter the immunological properties (both antigenicity and allergenicity) of an allergen: genetic variation of Der p 2 produces striking effects on T cell responses and IgE binding [38]. Regarding pollen allergens, recombinant Bet v 1 allergen shows reduced IgE binding compared with its highly allergenic Bet v 1a counterpart, from which only differs in nine residues [39,40]. On the opposite hand, recombinant rOle e 1/3c isoform of Ole e 1 [21] displays the highest degree of IgE-binding in comparison with rOle e 1/5c, rSir v 1 and rLig v 1 forms (the latest from *Syringa vulgaris *and *Ligustrum vulgare*, respectively), which present a progressively higher number of substitutions with respect to the amino acid sequence determined for Ole e 1 by Edman degradation [18-20]. Experimental evidence of the importance of certain positions of the amino acid sequence of Ole e 1 for both IgG and IgE binding and the elicitation of the allergenic response has been obtained since the nineties [21,24,25,41]. As the result of these studies, B-cell, T-cell epitopes and IgE and IgG B-cell immunodominant regions of Ole e 1 have been determined. It is foreseeable that the described substitutions affecting these regions will have a particular contribution in putative changes in the allergenicity/immunogenicity of the protein.

Furthermore, the induction of minor changes and the study of variants within an allergen is a commonly proposed approach to the engineering of hypoallergens. In accordance with the results showed here, some modified allergens, or even hypoallergenic forms of Ole e 1 could naturally occur in some olive varieties. An important evidence of this approach has recently emerged after the description of the generation and further immunological testing of three hypoallergenic mutants of Ole e 1 [42]. Two out of the three mutants generated by site-directed mutagenesis of Ole e 1-specific cDNA and produced in *Pichia pastoris *(135Δ10 and 140Δ5 deletion mutants) were able to strongly reduce the IgE-binding capability of sera from olive pollen-allergic patients. In addition, the 135Δ10 mutant was able to maintain intact its T cell reactivity and to induce blocking antibodies. From the sequences analyzed in the present paper, those three corresponding to Bella de España showed point substitutions in 135Y, and five sequences (three from Bella de España, one from Picual, and one from acebuche) displayed substitutions throughout the 144N and the 145M positions. On the basis of this coincidence in the altered regions of Ole e 1, the allergen isoforms of Bella de España could be considered as potential hypoallergenic forms of natural origin. Experimental data obtained in our laboratory have shown that the Ole e 1 variants from Bella de España pollen display reduced binding to IgEs from olive-allergic patients in Western blotting experiments, when compared to Ole e 1 forms from other olive cultivars (unpublished results). Future analysis of epitope reactivity should include putative modifications in other immuno-relevant regions of the protein which should also comprise the N-terminus of Ole e 1, whose sequence has not been analyzed in this work.

The presence of differences in the reactivity to Ole e 1 displayed by patients from different geographical origin in Spain [3,43,44] could be explained by the presence of Ole e 1 polymorphism linked to the varietal origin of the pollen present in the atmosphere, as the major olive varieties are precisely and discretely distributed over geographic regions [45]. A similar finding has been described for major house dust mite allergens, where the predominant variants of Der p 2 have been found to be distinct in different regions [26].

The analysis of the sequences showed here indicates that the Ole e 1 sequences from varieties like Bella de España display a higher degree of identity with Ole e 1-like proteins from *Oleaceae *than with the "canonical" sequence of Ole e 1 obtained after Edman degradation [20] or other Ole e 1 sequences described in the literature or present in databases. Important implications of such relationships, particularly regarding the presence of cross-reaction phenomena are expected: i.e. the exposure to pollen from such olive varieties (mainly anemophilous, and therefore highly represented like an aeroallergen in broad areas) may spread, or even trigger patient's reactivity to pollens from mainly entomophilous, and only locally represented plants species with low frequency of allergic sensitization like *Ligustrum*, *Fraxinus*, *Syringa *and other *Oleaceae *[46,47].

A large variety of morphological, biometric, biochemical and molecular characters have been widely used to describe and characterize olive germplasm [48]. Molecular markers include RFLPs, RAPDs, AFLPs, micro- and mini-satellite sequences. The low level of intra-varietal polymorphism showed by Ole e 1 sequences, compared to the higher polymorphism present among cultivars could be derived to the use of allergen sequences as molecular marker for olive breeding purposes. As an example, Ole e 1 sequences could be used as an additional marker in order to complement the discrimination between closely related olive cultivars, where other markers produce no or little differential patterns. The present study shows how two very closely related cultivars are recognized like different, on the basis of Ole e 1 similarity. This is the case of cultivars Picholine marocaine and Menara, the later considered a clonal selection of the former, raised in Morocco on the basis of better production, sooner come into bearing, and easier propagation from cuttings [49]. The increasing number of molecular markers available will help to elucidate important questions regarding the olive culture, which remain unanswered. Conflicting hypotheses exist about the phylogenetic relationships between *Olea europaea *L. and *Olea*-related species, between wild and cultivated forms of *Olea europaea *L. and about the origin of the culture [50]. Other biological implications of the allergen polymorphism in olive pollen with regard to pollen production and performance have been recently addressed [51].

Although much effort must yet be put into the characterization of olive pollen allergen polymorphism in relationship to the varietal origin, the advantages of the knowledge generated are obvious, and a number of applications are foreseeable. The study of natural variants will lead to the development of methods for improving efficacy and safety of the extracts currently in use for diagnostic and therapeutic purposes, as well as the routine trials for quality control of many laboratories. New strategies for the design of hypoallergenic variants will be considered and natural pollen extracts from different varietal sources could be used in order to further personalize patient's reactivity or like natural hypoallergenic extracts, as an alternative or in addition to the use of recombinant allergens. Finally, advances in the breeding of hypoallergenic cultivars might also be directed by a broader knowledge of the genetic basis of this variability. The clinical implications of allergen polymorphism in the olive pollen are examined in detail in [51], in particularly how cultivar differences may affect extract quality, diagnostic and therapeutic efficacy and safety, and the development of new vaccines.

## Conclusion

The sequence polymorphism present in the olive pollen major allergen Ole e 1 is highly dependent on the genetic origin of the pollen analyzed. Predicted Ole e 1 proteins from different olive cultivars are likely to display different qualitative properties. These differences have a number of implications in olive pollen biology. They also should be considered for optimal design of allergen formulations, including the design of recombinant allergens, in order to improve both their efficiency and safety.

## Methods

### Plant material

Olive (*Olea europaea *L.) pollen was individually collected during May and June 2002–2005 from selected olive trees of cultivars Lucio, Picual, Loaime, Hojiblanca, Arbequina and Bella de España (Olive World Germplasm Bank, Córdoba, Spain), and Picholine marocaine and Menara (olive collection of Ain Taoujdat, Meknes, Morocco). Pollen samples were collected in large paper bags by vigorously shaking the inflorescences, sequentially sieved through 150 and 50 μm mesh nylon filters to eliminate debris and maintained at -80°C.

### Amplification and cloning of Ole e 1 transcripts

RT-PCR procedures were used as described by [17] with minor modifications. Briefly, total RNA was isolated from mature pollen of each cultivar using an RNeasy Plant Total RNA kit (Quiagen). cDNA synthesis was carried out by using M-MuLV reverse transcriptase (MBI Fermentas) and a poly-dT RACE adaptor (5'-GACTCGAGTCGACATCGA(T)_17_-3') as a primer, following manufacturer's indications. PCR amplifications were carried out from 250 ng of the template cDNAs, by using 5 ρ mol of the primer (5'-ACCTCCAGTTTCTCAATTTCAC-3') as forward and equal amount of the poly-dT RACE adaptor described above. The mixtures were denatured at 95°C for 3 min and subjected to PCR amplification in a Progene Thermocycler (Techne) for 30 cycles of 94°C for 1 min, 57°C for 1 min and 72°C for 1 min, using Pfu polymerase (Stratagene). After analyzing the PCR products by gel electrophoresis, the bands obtained (650 bp) were cloned into pGEM-T Easy vector (Promega), according to the manufacturer's instructions. At least three clones from each cultivar were sequenced.

### Bioinformatic analysis of Ole e 1 sequences

Nucleotide sequences were analyzed by the nucleotide-nucleotide BLAST (blastn) program [23]. BLAST pairwise alignments and tree view were generated by using the fast minimum evolution method and maximum sequence difference of 0.75.

Protein sequence alignments were performed by the ClustalW software [52] and viewed using the Jalview viewer 2.2 [53]. Conservation index was calculated as described by [54]. Ole e 1 protein sequences were searched for different motifs with the ScanProsite on-line facilities at the ExPASy Proteomics Server [55].

## Authors' contributions

JDA and MIRG designed the study. AMHK and AJC designed the experiments. AMHK, AJC and JCJL cloned and obtained the sequences. JDA and AMHK analyzed the sequences. JDA, MIRG and AMHK prepared the manuscript.

## Supplementary Material

Additional file 1 nucleotide sequences alignment. Multiple alignment of the Ole e 1 nucleotide sequences displayed in Fig 1. Start and stop codons (when available) are indicated by boxes. Note the absence of a 38 bp fragment in the sequences corresponding to the cultivar Bella de España. Numbering begins at the ATG start codon. Click here for file

Additional file 2 Comparison of nucleotide and deduced amino acid sequences of the different forms of Ole e 1 and Ole e 1-like proteins presented in Figs 1 and 2. The cells of the upper right section show the calculated percentage of identity between nucleotide sequences. Cells of the lower left section show the calculated percentages of identity between amino acid sequences in absolute terms, or after taking into account non-conservative changes only. Click here for file

Additional file 3 Key amino acid substitutions throughout the Ole e 1 and Ole e 1-like sequences analyzed. Key substitutions analyzed include: conserved Cys residues, an extra site for potential N-glycosylation, point substitutions in those motifs described like immunodominant T-cell epitopes of Ole e 1 allergen [24], and amino acid residues implicated in IgG and IgE binding [25]. Deduced sequences fully identical to the canonical sequence of Ole e 1 obtained after Edman degradation [20] are marked by a double asterisk. Click here for file
