# Supplementary figures and images for: Olive cultivar origin is a major cause of polymorphism for Ole e 1 pollen allergen
Source: BMC Plant Biol. 2008 Jan 25;8:10. doi: 10.1186/1471-2229-8-10 (PMC2275730; doi:10.1186/1471-2229-8-10)

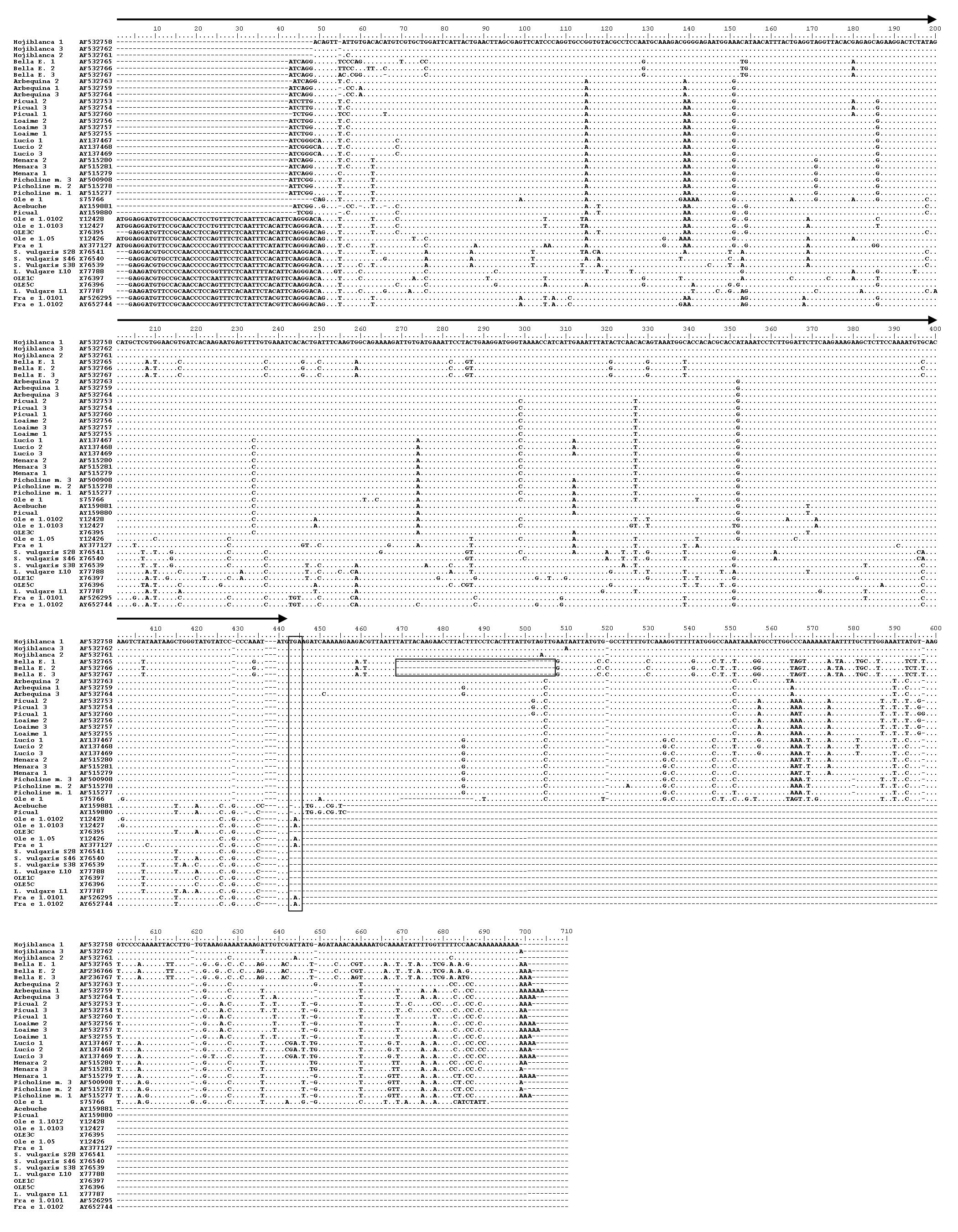

Supplement: Additional file 1 — nucleotide sequences alignment. Multiple alignment of the Ole e 1 nucleotide sequences displayed in Fig 1. Start and stop codons (when available) are indicated by boxes. Note the absence of a 38 bp fragment in the sequences corresponding to the cultivar Bella de España. Numbering begins at the ATG start codon. [file 1471-2229-8-10-S1.JPEG]

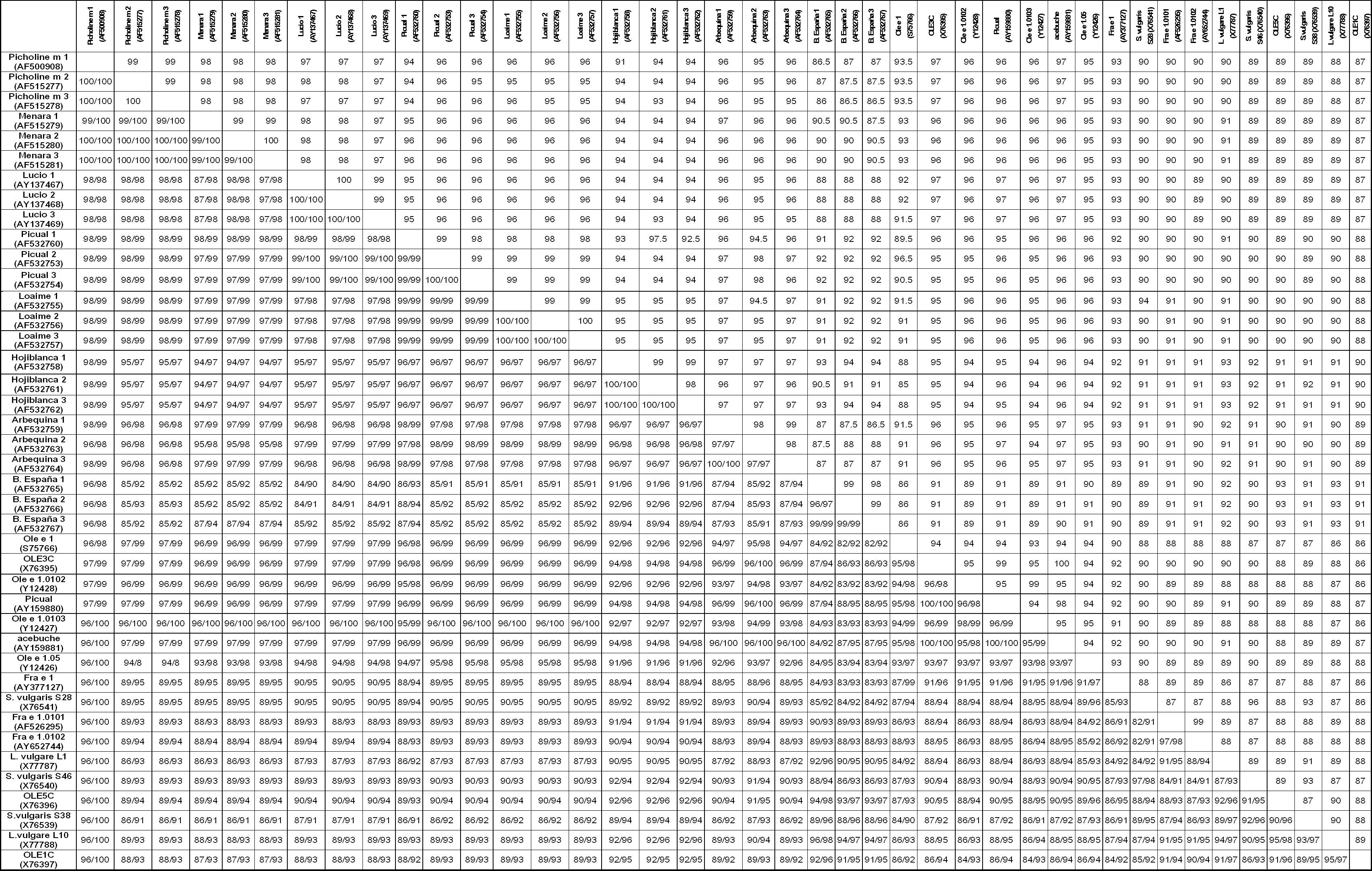

Supplement: Additional file 2 — Comparison of nucleotide and deduced amino acid sequences of the different forms of Ole e 1 and Ole e 1-like proteins presented in Figs 1 and 2. The cells of the upper right section show the calculated percentage of identity between nucleotide sequences. Cells of the lower left section show the calculated percentages of identity between amino acid sequences in absolute terms, or after taking into account non-conservative changes only. [file 1471-2229-8-10-S2.jpeg]

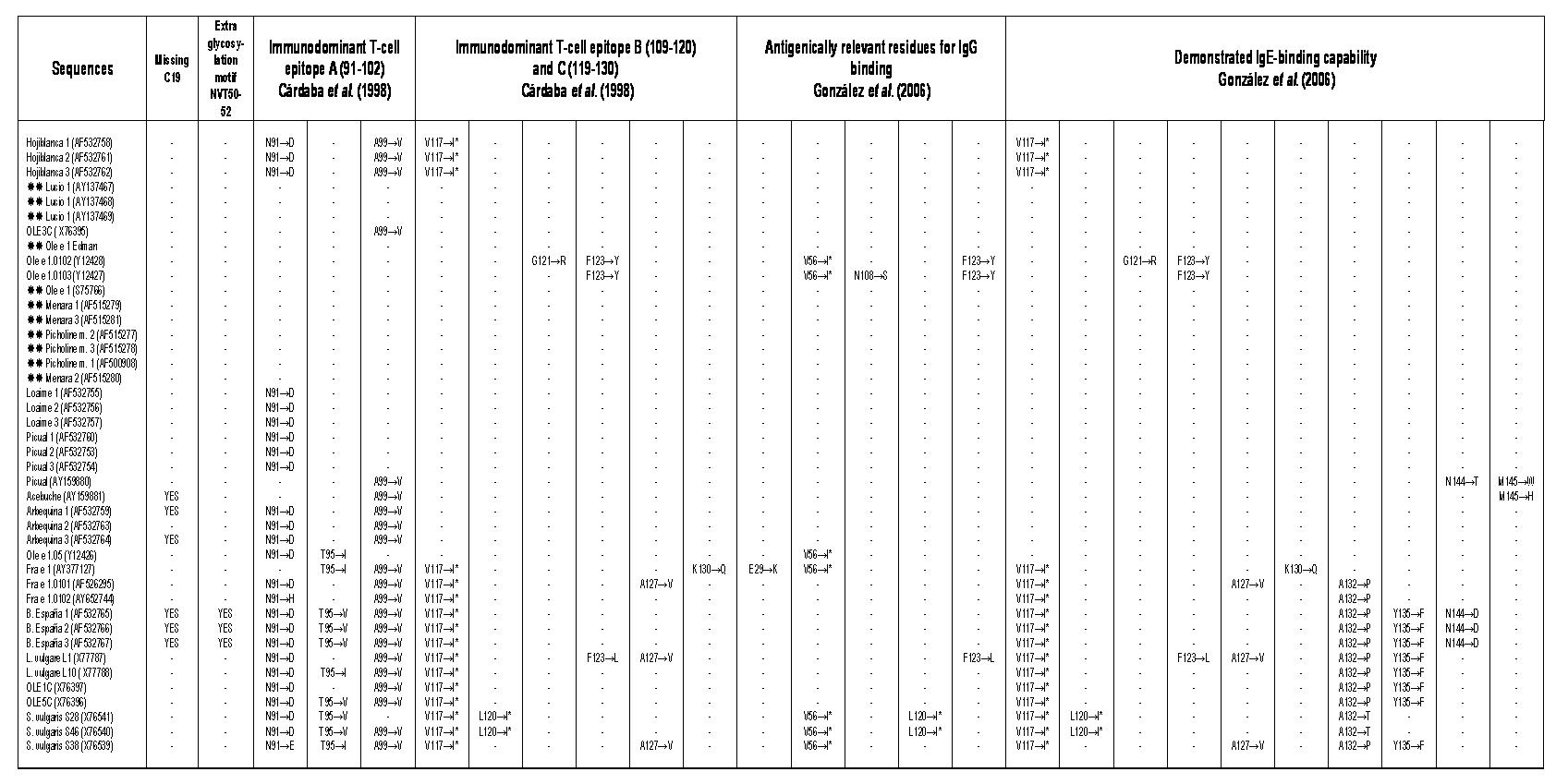

Supplement: Additional file 3 — Key amino acid substitutions throughout the Ole e 1 and Ole e 1-like sequences analyzed. Key substitutions analyzed include: conserved Cys residues, an extra site for potential N-glycosylation, point substitutions in those motifs described like immunodominant T-cell epitopes of Ole e 1 allergen [24], and amino acid residues implicated in IgG and IgE binding [25]. Deduced sequences fully identical to the canonical sequence of Ole e 1 obtained after Edman degradation [20] are marked by a double asterisk. [file 1471-2229-8-10-S3.jpeg]
